# Non-myogenic Contribution to Muscle Development and Homeostasis: The Role of Connective Tissues

**DOI:** 10.3389/fcell.2017.00022

**Published:** 2017-03-23

**Authors:** Sonya Nassari, Delphine Duprez, Claire Fournier-Thibault

**Affiliations:** Developmental Biology Laboratory, IBPS, Centre National de la Recherche Scientifique UMR7622, Institut National de la Santé Et de la Recherche Médicale U1156, Université Pierre et Marie Curie, Sorbonne UniversitésParis, France

**Keywords:** connective tissue, muscles, bones, tendons, development, regeneration

## Abstract

Skeletal muscles belong to the musculoskeletal system, which is composed of bone, tendon, ligament and irregular connective tissue, and closely associated with motor nerves and blood vessels. The intrinsic molecular signals regulating myogenesis have been extensively investigated. However, muscle development, homeostasis and regeneration require interactions with surrounding tissues and the cellular and molecular aspects of this dialogue have not been completely elucidated. During development and adult life, myogenic cells are closely associated with the different types of connective tissue. Connective tissues are defined as specialized (bone and cartilage), dense regular (tendon and ligament) and dense irregular connective tissue. The role of connective tissue in muscle morphogenesis has been investigated, thanks to the identification of transcription factors that characterize the different types of connective tissues. Here, we review the development of the various connective tissues in the context of the musculoskeletal system and highlight their important role in delivering information necessary for correct muscle morphogenesis, from the early step of myoblast differentiation to the late stage of muscle maturation. Interactions between muscle and connective tissue are also critical in the adult during muscle regeneration, as impairment of the regenerative potential after injury or in neuromuscular diseases results in the progressive replacement of the muscle mass by fibrotic tissue. We conclude that bi-directional communication between muscle and connective tissue is critical for a correct assembly of the musculoskeletal system during development as well as to maintain its homeostasis in the adult.

## Introduction

Skeletal muscle forms a highly complex and heterogeneous structure, which is part of the musculoskeletal system of the body. The process of generating muscles is defined as “myogenesis.” This mechanism occurring during development is an important step in the establishment of the musculoskeletal system allowing its essential functions, for instance body motion or the ability to breath. Myogenesis occurs through successive and overlapping phases that ultimately give rise to correctly patterned muscles. In the first phase of myogenesis, which is called embryonic myogenesis, embryonic progenitors cells form primary muscle fibers, which constitute the scaffold of the muscles. During the second phase of myogenesis named fetal myogenesis, fetal progenitors fuse between themselves and with primary fibers to form secondary fibers and allow muscle growth. Both waves of myogenesis occur during embryonic development, and involve specific types of muscle progenitors cells. After birth, a third wave of myogenesis can be activated during muscle regeneration, which occurs after muscle damage. This step involves muscle stem cells, so-called muscle satellite cells, which contribute to muscle reconstruction by fusing with the existing muscle fibers or generating new muscle fibers (Stockdale, 1992; Tajbakhsh, 2009; Tedesco et al., 2010). Studies suggest that embryonic myogenesis is largely exhausted at the end of embryonic development, while fetal and perinatal phases of myogenesis persist to contribute to the majority of adult muscle stem cells (reviewed in Tajbakhsh, 2009).

The intrinsic molecular signals regulating the different waves of myogenesis have been well described in the literature. However, the interactions between muscles and adjacent tissues during development are not completely elucidated. During development and adult life, as part of the musculoskeletal system, muscles are closely associated with the other components of this system: bone, cartilage, tendon, ligament and irregular connective tissue, all of them emerging from the family of connective tissues. Although the interactions between the different components of the musculoskeletal system during development has been highlighted from the 1980's, more recent work has begun to decipher the molecular mechanisms underlying the importance of connective tissue in the regulation of developmental and regenerative myogenesis.

The scope of this review is to synthesize the data supporting the process of connective tissue-mediated muscle development and regeneration and to point out the active role of this so-called “supporting tissue” in muscle formation and repair. Indeed, defect in connective tissue-muscle interactions can lead to human pathology, as congenital diaphragmatic hernias, a birth defect of the diaphragm muscle (Merrell et al., 2015), or the Holt-Oram syndrome characterized by skeletal defects of the upper limbs and heart anomalies (Hasson et al., 2010). In addition, in skeletal muscle regenerative disorders (muscular dystrophies) as well as in aging (sarcopenia), the impairment of the muscle regenerative potential correlates with a progressive replacement of contractile mass by fibrotic and adipose tissues (reviewed in Farup et al., 2015). It is therefore necessary to better understand the interactions occurring between the different components of the musculoskeletal system. This would allow us to decipher the molecular mechanisms underlying muscle disorders not related to the impairment of intrinsic regulation of myogenesis.

## Connective tissue development

### Different types of connective tissues

In the body, the main role of **connective tissues (CTs)** is to support and connect organs together. CTs are primarily composed of fibroblasts and extracellular matrix consisting of amorphous gel-like and matrix fibers. The amorphous gel-like, named ground substance, mostly contains glycoproteins and proteoglycans, while the fibrous network is made of collagen and elastic fibers (Omelyanenko and Slutsky, 2013). Among the **supportive** CTs, two main types can be distinguished: the **specialized CT** and the **dense CT**. The specialized CT refers to bones and cartilage elements. The dense CT is further divided into two subtypes: the **dense regular CT** and the **dense irregular CT**, which refer respectively to tendon/ligament structures and to CT embedding organs (Table 1). The nature and function of these different CTs are predominately determined by the composition and organization of the extracellular matrix. In dense regular CT, fibroblasts produce a significant amount of collagen fibers that display a spatial organization, while in the dense irregular CT, fibroblasts produce collagen fibers that do not present any specific organization (Omelyanenko and Slutsky, 2013).

**Table 1:** **Classification of the different types of connective tissues**.

**Connective tissue types**			
**Proper**			**Specialized**
**Soft**	**Dense**		
**Adipose tissue**: brown, beige and white adipose tissue **Areolar** tissue: sub-cutaneous, around blood vessels, and nerves **Reticular tissue**: into the liver, pancreas, lymph nodes, spleen, bone marrow	**Regular**	**Irregular**	**Bones cartilage**
	**Tendons:** direct tendons, wrap-around tendons **Ligaments:** intra articular and extraarticular, synovial joints	Dermis Capsules of organs (periosteum, epimysium) Walls of tubular organs Muscle connective tissue (endomysium, perimysium)	

### Connective tissue formation

**Figure 1:**
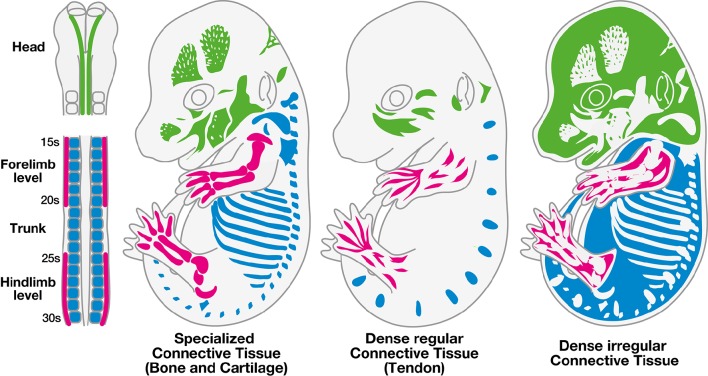
**Embryonic origins of the different types of connective tissues**. The different types of connective tissues, specialized (bone and cartilage), dense (tendon) and irregular connective tissues are depicted in three mouse E14.5 embryos. The color code corresponds to the embryological origins of the different types of connective tissues, which differ depending on their location in the body. Connective tissues of the head derive from neural crest cells (green), while trunk connective tissues arise from the somites (blue) and limb connective tissues arise from the lateral plate mesoderm (pink).

**Figure 2:**
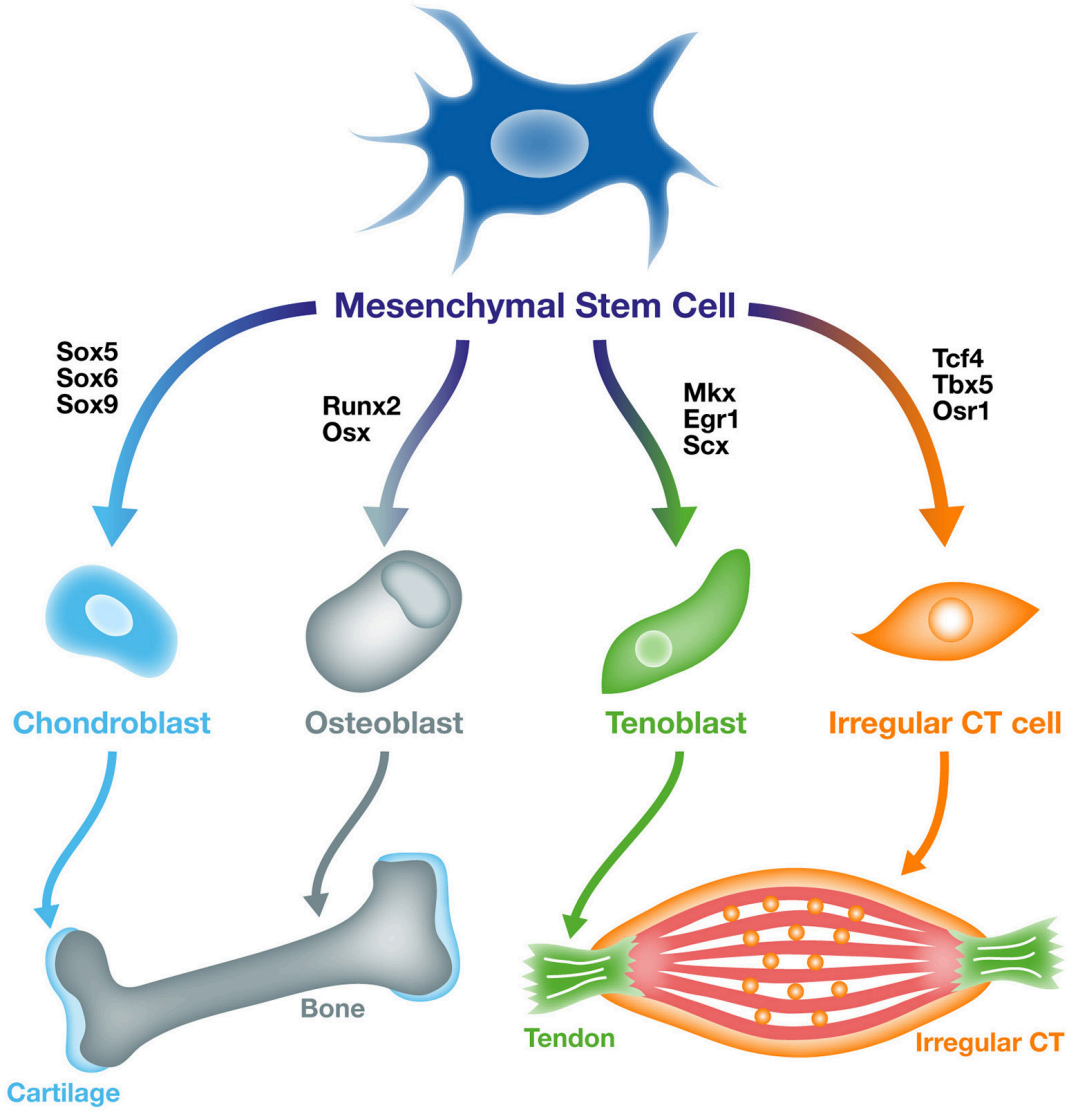
**From mesenchymal stem cells to specific connective tissue cell types**. Undifferentiated mesoderm-derived cells or mesenchymal stem cells are able to differentiate into different types of connective tissues including, bone, cartilage, tendon, and irregular connective tissue. Specific transcription factors have been identified as able to induce mesenchymal stem cell differentiation toward the different types of connective tissue cells. The *Sox5/6/9, Runx2/Osx, Scx/Mkx/Egr1*, and *Tcf4/Tbx5/Osr1* genes drive undifferentiated cells to differentiate into cartilage, bone, tendon and irregular connective tissue, respectively.

During embryonic development, undifferentiated mesenchymal cells, derived from mesodermal and mesectodermal (neural crest cells) origins, give rise to the different forms of CT: bones, cartilage, tendons, ligaments, and irregular CT (Wachtler et al., 1981). Head CTs originate from neural crest cells, while CTs of the body originate from paraxial or lateral plate mesoderm (Figure 1). The specification and differentiation processes of the different types of CTs is controlled by specific key transcription factors or signaling molecules. Irrespective to their embryological origins, transcription factors have been identified for the specification of each type of CT from undifferentiated mesenchymal cells (Figure 2).

#### Specialized connective tissue (bone and cartilage)

The embryonic origins of cartilage and bone are multiple. Indeed, elements of the trunk, head and limb skeleton arise from three distinct embryonic structures, somites, neural crest cells and lateral plate mesoderm (Figure 1, Wachtler et al., 1981; Christ and Wilting, 1992; Noden and Trainor, 2005). The process of skeleton formation, which corresponds to the development of cartilage and bone elements, is initiated by the condensation of undifferentiated mesenchymal cells at the future sites of bones. Following condensation, mesenchymal precursors undergo either chondrocyte or osteoblast differentiation, giving rise respectively to cartilage or bone. Osteogenesis characterizes the process of ossification, which occurs through two different mechanisms. The process of intramembranous ossification corresponds to a direct transition from condensed undifferentiated-mesenchymal cells into osteoblasts (as described above). The second mechanism of bone formation is called endochondral ossification. It defines the replacement of cartilage by bone. In this case, chondrogenesis is the first step in a process that ultimately gives rise to bones. Intramembranous ossification occurs in bones of the skull, while other bones form by endochondral ossification (reviewed in Karsenty and Wagner, 2002).

Molecular mechanisms involved in cartilage and bone specifications are well understood (Figure 2). Members of the SOX (SRY-related HMG-box) transcription factor family are key players in the regulation of cartilage specification (Lefebvre et al., 1998). During mouse embryonic development, *Sox9* presents a similar expression pattern to *Col2a1*, the main collagen in the cartilage extracellular matrix (Zhao et al., 1997). In mouse mutant embryos for *Sox9*, cartilage development fails. The complete absence of cartilage elements in *Sox9* mutant mice highlights a role for *Sox9* in the regulation of mesenchymal cell condensation and differentiation toward a cartilage fate (Bi et al., 1999; Akiyama et al., 2002). Moreover, it has been shown that *Sox9* is required for the expression of two additional *Sox* genes, *Sox5*, and *Sox6* that are co-expressed with *Sox9* in committed cartilage cells (chondrocytes), (Lefebvre et al., 1998, 2001). Both *Sox5* and *Sox6* mutant mice show skeletal abnormalities, with no modification of *Sox9* expression, demonstrating that *Sox9* acts upstream of *Sox5* and *Sox6* (Smits et al., 2001).

*Runx2* (Runt-related transcription factor 2) is a master gene for osteogenesis (Komori et al., 1997; Ducy et al., 1999). This transcription factor is specific to bone progenitor cell lineage (Ducy et al., 1999). Knockout mice for *Runx2* show no osteogenesis. While cartilage elements are still present in *Runx2* ^−/−^ mouse, all bones are missing, demonstrating the importance of *Runx2* in bone specification (Komori et al., 1997). In contrast to *Sox9*, which is required for cartilage differentiation in addition to specification (Akiyama et al., 2002), *Runx2* is not required for bone differentiation (Takarada et al., 2013). After osteogenic cell commitment, *Runx2* activity has to be shut down to allow immature committed bone cells to become fully mature and to differentiate (Yoshida et al., 2004; Takarada et al., 2013; Adhami et al., 2014). *Osterix* (*Osx*) is also a key transcription factor in bone formation (Nakashima et al., 2002). *Osx* is specifically expressed in all bones (Nakashima et al., 2002) and is required for differentiation of bone progenitor cells. In mutant mouse for *Osx*, no bone is observed, however *Runx2* expression is maintained (Nakashima et al., 2002). Conversely, *Osx* expression is absent in Runx2 mutant mice (Nakashima et al., 2002). This indicates that *Runx2* and *Osx* are involved in bone specification and differentiation, respectively.

Beside the specific transcription factors, major signaling pathways have also been demonstrated to be involved in skeletal development. Wnt pathway regulates skeleton differentiation through a cell-autonomous mechanism, which enhances osteoblast differentiation at the expense of chondrocytes (Day et al., 2005; Hill et al., 2005). Conditional inactivation of ß-catenin in mesenchyme blocks osteoblast differentiation and induces ectopic chondrocytes (Day et al., 2005). In addition, ß-catenin has been shown to control the expression of *Sox9* and *Runx2 in vitro* (Day et al., 2005). The role of FGF signaling in skeletal development comes from the observations that *FgfR3* and *FgfR1* inactivation in mouse leads to achondroplasia and hypochondroplasia (Deng et al., 1996; Jacob et al., 2006). In both mutant mice, an expansion of the hypertrophic chondrocyte zone is observed, suggesting that FGF signaling is a negative regulator of chondrocyte proliferation (Deng et al., 1996; Jacob et al., 2006). Inactivation of one member of the Hedgehog family, *Ihh* (Indian Hedgehog), leads to a decrease in chondrocyte proliferation and a defect in osteoblast formation (Vortkamp et al., 1996). This effect is mediated by the interaction between Ihh and Parathyroid hormone, which maintains the rate between cell proliferation and differentiation (Kronenberg, 2006). Interestingly, RUNX2 induces *Ihh* expression which inhibits *Runx2* expression by a feedback loop mechanism (Yoshida et al., 2004). Finally, BMPS (Bone Morphogenetic Proteins) are important regulators of chondrocyte differentiation (Kobayashi et al., 2005, reviewed in Li and Cao, 2006) and have been shown to regulate *IHH* expression in chick embryos (Zhang et al., 2003).

#### Dense regular connective tissue (tendon)

Similarly to specialized CTs (bone and cartilage), tendons arise from distinct embryological origins depending on their position in the body (Figure 1). Tendons of the trunk originate from somites, more precisely from a subregion of the sclerotome named the syndetome (Brent et al., 2003), tendons of the craniofacial region derive from neural crest cells (Crane and Trainor, 2006; Grenier et al., 2009) and limb tendons derive from the lateral plate mesoderm (Kieny and Chevallier, 1979). Tendons attach muscles to bones by connecting muscle at the myotendinous junction and connecting bone at the enthesis, while ligaments connect bone to bone. The role of tendons is to transmit forces generated by muscle contractions to bones, in order to allow joint movements and maintain articular stability. The tendon extracellular matrix is rich in type I collagen fibers, which display a specific spatial organization parallel to the tendon axis. This specific organization lends mechanical properties to tendons (Benjamin and Ralphs, 2000). Ligaments are essential components of the skeletal joints. Their elasticity defines the range of motion of the joints, supports joint stability and protects joints and bones by their stretching capacities. Tendons and ligaments display similar structural collagen organization and molecular markers (Benjamin and Ralphs, 2000). However, genome-wide analysis identifies different levels of gene expression between adult tendons and ligaments (Pearse et al., 2009). However, tendon development has been more studied than ligament development (Tozer and Duprez, 2005).

In contrast to cartilage and bone, the master gene(s) involved in tendon specification during development is (are) still unknown. To date, *Scx* (Scleraxis) is the unique early tendon marker that has been described in vertebrates. *Scx* is specifically expressed in tendon progenitors and differentiated cells (Schweitzer et al., 2001). *Scx* mutant mice display severe tendon defects, leading to a severe impairment of limb and tail force-transmitting tendons, while anchoring tendons are less affected (Murchison et al., 2007). However, tendon progenitors are still present in *Scx*^−/^^−^, indicating that *Scx* is not the master gene driving tenogenesis during development. Two additional transcription factors have been identified to be involved in tendon formation, the homeobox transcription factor *Mkx* (Mohawk), (Ito et al., 2010; Liu et al., 2010) and the zinc finger transcription factor *Egr1* (Early Growth Response 1), (Lejard et al., 2011, Figure 2). Both *Mkx* and *Egr1* mutant mice display tendon defects associated with a decrease in *Col1a1* expression levels and in type I collagen fiber number in tendons (Ito et al., 2010; Liu et al., 2010; Lejard et al., 2011; Guerquin et al., 2013). However, both *Mkx* and *Egr1* are expressed after *Scx* during development and are not specific to tendons, since they are expressed in many other lineages (Rackley et al., 1995; Anderson et al., 2006).

TGFß (Transforming growth factor) and FGF (Fibroblast growth factor) signaling pathways have been shown to regulate tendon specification and differentiation at different places of the body (recently reviewed in Gaut and Duprez, 2016). As mentioned above, **axial** tendon progenitors arise from a somitic subcompartment named the syndetome. The syndetome compartment, localized at the interface between the sclerotome and myotome, is formed by *Scx*-expressing cells. In chick embryos, axial tendons do not develop in the absence of axial muscles, as demonstrated by the absence of tendons after dermomyotome removal (Brent et al., 2003). Chick axial *SCX* expression is induced in response to FGF signaling arising from the myotome, which concomitantly downregulates *PAX1* expression in the sclerotome (Brent et al., 2003). In contrast to axial tendons, the initiation of **head and limb** tendons is independent of muscle. In the absence of limb or craniofacial muscles, *Scxa/SCX/Scx* expression is normally induced in limb and head of zebrafish, chick and mouse embryos (Schweitzer et al., 2001; Edom-Vovard et al., 2002; Grenier et al., 2009; Chen and Galloway, 2014). In chick limbs, *SCX* induction is known to be mediated via ectodermal signals, as shown by the absence of *SCX* expression after ectoderm removal (Schweitzer et al., 2001). BMP signaling from the limb mesenchyme represses *SCX* expression and overexpression of the BMP antagonist NOGGIN leads to ectopic *SCX* expression, indicating that tendon specification in chick limbs results from a balance between an unidentified factor coming from the ectoderm and BMP signaling from the mesenchyme (Schweitzer et al., 2001). TGFß is a key signaling molecule for tendon development. TGFß signaling is required and sufficient for *Scx/SCX* expression during development in chick and mouse embryos (Pryce et al., 2009; Havis et al., 2014, 2016), while FGF signaling is required and sufficient for *SCX* expression in undifferentiated chick limb cells but not in mouse limb cells (Pryce et al., 2009; Havis et al., 2014, 2016).

Although muscles are not necessary for head and limb tendon initiation, they are required for the maintenance of *Scxa/SCX/Scx* expression in tendons and for full tendon differentiation. In the absence of muscles, tendons degenerate in chick, mouse and zebrafish embryos (Kardon, 1998; Edom-Vovard et al., 2002; Grenier et al., 2009; Chen and Galloway, 2014). Moreover, overexpression of FGF4, which is normally expressed at the tips of muscles fibers, leads to ectopic expression of tendon-associated genes in chick limbs (Edom-Vovard et al., 2002; Eloy-trinquet et al., 2009). In addition, chick embryo immobilization decreases *SCX* expression in limb tendons and application of FGF4 or TGFβ ligands prevents *SCX* down-regulation consecutive to immobilization, demonstrating that FGF and TGFβ act downstream of mechanical forces to regulate tendon differentiation (Havis et al., 2016).

#### Dense irregular connective tissue

The irregular CT constitutes a protective envelop for the different organs of the body, by embedding and scaffolding organs, with scattered cells embedded in high extracellular matrix content. Irregular CT is present all around organs, but also inside organs. First studies on the differentiation of irregular CT have focused on the extracellular matrix composition. During development, Type I and type III collagen are both expressed in dense regular and irregular CTs, however type I collagen tends to replace type III collagen in adult tendons, while mature irregular CT is characterized by the expression of both type III and type VI collagen (Kieny and Mauger, 1984; Zhang et al., 2005; Gara et al., 2011; Stricker et al., 2012). Due to the lack of specific early molecular markers, the mechanisms driving irregular CT specification have been poorly investigated. However, the recent identification of transcription factors expressed in irregular CT has provided new insights into irregular CT formation and function (Figure 2).

The first marker identified in irregular CT fibroblasts is the transcription factor TCF4, belonging to the TCF/LEF family. In limbs of both mouse and chick embryos, *Tcf4*-expressing cells discriminate the lateral plate-derived mesodermal population from myogenic cells (Kardon et al., 2003; Mathew et al., 2011). When chick limb muscles differentiate, *TCF4* expression is restricted to muscle CT (Kardon et al., 2003) and colocalizes with type I collagen. Expression of *TCF4* in muscle CT persists at adult stages (Mathew et al., 2011). *TCF4* misexpression in chick limbs leads to muscle patterning defects, highlighting a non-cell autonomous effect of muscle CT on muscles, in which TCF4-expressing fibroblasts define a pre-pattern that ultimately drive muscle patterning (Kardon et al., 2003). However, low levels of *Tcf4* have been also observed genetically in myogenic cells (Murphy et al., 2011). BMP signaling has been shown to negatively regulate *TCF4* expression (Bonafede et al., 2006), while Wnt signaling positively regulates *TCF4* expression (Kardon et al., 2003) in developing chick limbs. *TCF4* is also expressed dynamically in avian jaw muscle CT and has been shown to be regulated by neural crest mesenchyme (Tokita and Schneider, 2009).

The T-box transcription factor *Tbx5* is another gene that has been characterized as expressed in fibroblasts constituting irregular CT. At early stage of mouse limb bud development (E11.5), *Tbx5* is broadly expressed in lateral plate mesodermal cells in domains overlapping with bone, tendon and muscle progenitors (Hasson et al., 2007). Disruption of *Tbx5* function in mice leads to disorganization of muscle CT during embryonic development (Hasson et al., 2010), which could be related to subtle alterations of muscle CT markers, as *Tcf4* and the *Osr* genes (see below), (Hasson et al., 2010). *Tbx5* positively regulates the expression of N-cadherin and ß-Catenin in muscle CT and as the expression levels of Wnt signaling targets are not affected in *Tbx5* mutant, it seems noteworthy that *Tbx5* mostly affects cell adhesion mechanisms independently of *Tcf4*.

Two orthologs of the Odd-Skipped genes, *Osr1* and *Osr2*, has been described as expressed in the irregular CT in chick and mouse embryos (Stricker et al., 2006, 2012). Both genes are expressed in a variety of organs such as kidney, eye, branchial arches, and dermis (So and Danielian, 1999; Lan et al., 2001; Stricker et al., 2006). In the developing limb of mouse and chick embryos, *Osr1* is expressed in all irregular CTs, displaying a partial overlap with *Tcf4* (Stricker et al., 2006). *Osr2*, although widely expressed in irregular CT, shows prevalence for muscle CT (Stricker et al., 2006, 2012). Both genes are also expressed in the mesenchyme of branchial arches in chick (Stricker et al., 2006) and mouse (Liu et al., 2013) embryos. Forced expression of OSR1 or OSR2 in chick mesenchymal progenitor limb cells induces the expression of irregular CT markers such as *COL3A1* and *COL6A1* and down-regulates the expression of markers of cartilage (specialized CT) and tendon (dense regular CT), (Stricker et al., 2012). Conversely, *OSR1* or *OSR2* inactivation down-regulates *COL3A1* and *COL6A1* expression, while increasing cartilage formation in chick limb cells (Stricker et al., 2012). Similarly, specific inactivation of *Osr1* in cranial neural crest cells result in the formation of an ectopic cartilage in the developing mouse tongue (Liu et al., 2013). OSR1 has been shown to bind *Sox9* promoter and repress *Sox9* expression, indicating that OSR1 prevents chondrogenesis in the mammalian tongue through repression of *Sox9* expression (Liu et al., 2013).

## Muscle development

### Embryonic origins of skeletal muscles

**Figure 3:**
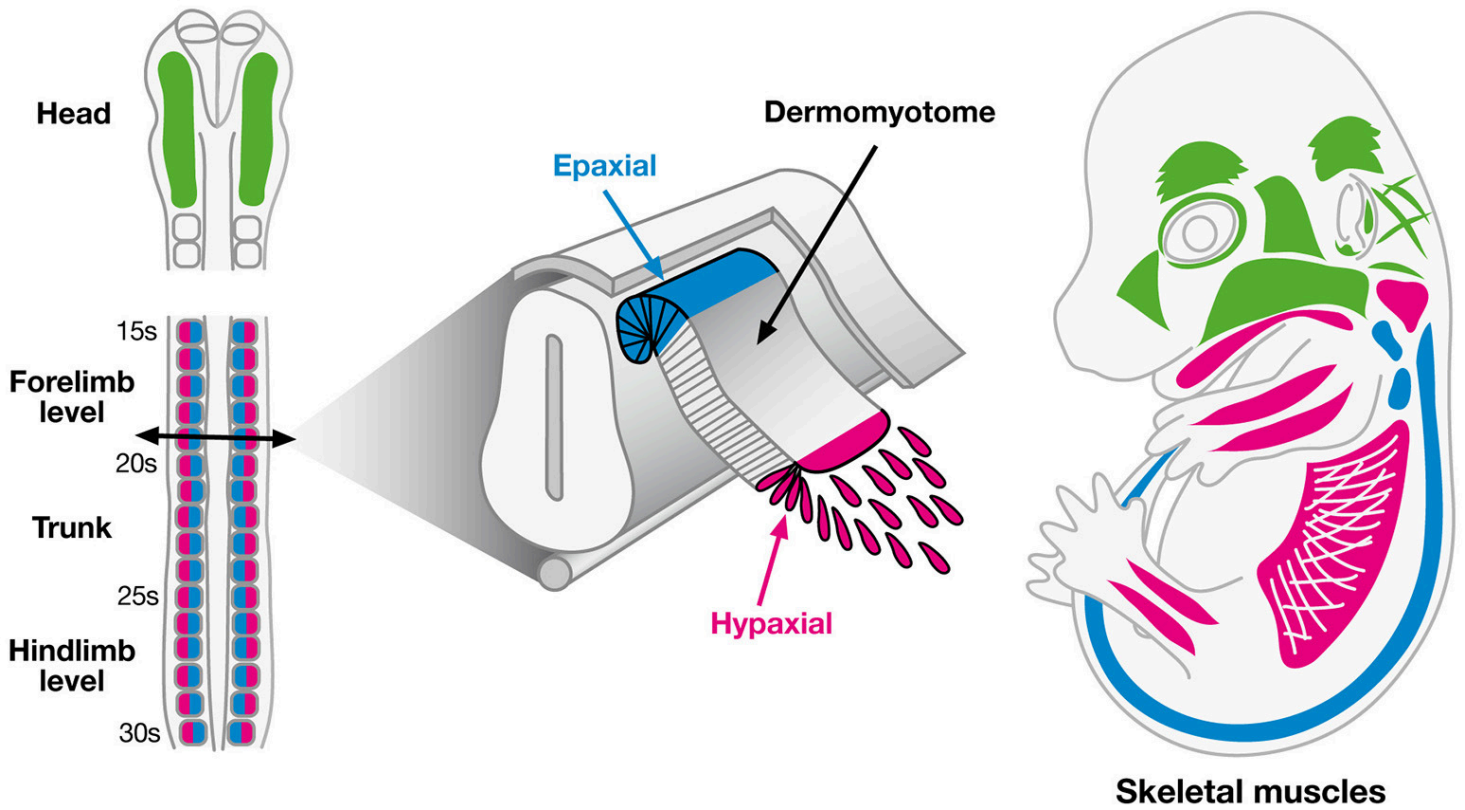
**Embryonic origins of skeletal muscles**. Myogenic cells of skeletal muscles have two distinct embryonic origins. Myogenic cells of head muscles originate from the paraxial mesoderm (green), except the tongue and posterior neck muscles, which originate from the hypaxial lip of dermomyotome of cranial somites (pink). In the trunk, myogenic cells of back muscles derive from the epaxial lip of dermomyotome (blue), while myogenic cells of diaphragm and limb muscles derive from the hypaxial lip of dermomyotome (pink).

In vertebrates, all skeletal muscles derive from paraxial mesodermal cells (Figure 3; reviewed in Stockdale et al., 2000; Noden and Francis-west, 2006), with the exception of a small population of neck muscles that have been shown to derive from the lateral plate mesoderm (Theis et al., 2010). Most of the knowledge about the paraxial mesodermal origin of skeletal muscles was established thanks to Di-I labeling (Selleck and Stern, 1991) and chick-quail graft experiments (Couly et al., 1992; Ordahl and Le Douarin, 1992). These lineage studies showed that although skeletal muscles share a common mesodermal origin, muscle organization significantly differs depending on their rostro-caudal position in the embryo.

Head muscles originate from cranial paraxial mesoderm. Cranial paraxial mesoderm lacks any initial signs of segmentation and mesodermal cells will only be segregated once they reach the branchial arches concomitantly with cranial neural crest cells (Figure 3; reviewed in Noden and Francis-west, 2006). Three distinct groups of cranial muscles can be distinguished: the extraocular muscles, originating from the prechordal mesoderm, the branchiomeric muscles including the muscles of the jaw, anterior neck and face, arising from the paraxial mesoderm and the tongue and posterior neck muscles, deriving from anterior somites (Noden, 1983; Couly et al., 1992; Trainor et al., 1994).

Truncal paraxial mesoderm caudal to the head emerges from already segmented embryonic structures, the somites, that will give rise to two main compartments all along the truncal axis of the embryo, the sclerotome and the dermomyotome (Figure 3, reviewed by Christ and Ordahl, 1995). Limb and axial skeletal muscles originate from the dermomyotome. The dorsomedial part of the dermomyotome gives rise to the epaxial musculature corresponding to the back and intercostal muscles, while the ventrolateral part of the dermomyotome gives rise to the hypaxial musculature corresponding to the diaphragm, abdominal and limb muscles (Ordahl and Le Douarin, 1992). Few muscles from the most posterior part of the head, including tongue muscles and muscles of the posterior pharyngeal arches also develop from the somites (Noden and Francis-west, 2006).

### Molecular cascades that regulate muscle development

**Figure 4:**
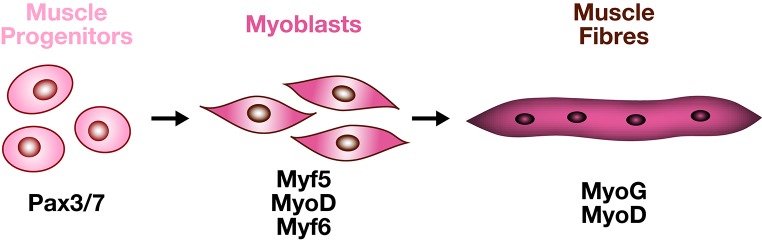
**From muscle progenitors to muscle fibers**. The myogenic program is characterized by the successive and overlapping expression of specific transcription factors. PAX3 and PAX7 label the progenitor state. The MYF5, MYF6, MYOD myogenic factors label the entry into the myogenic program, MYOG is characteristic of differentiated multinucleated muscle cells.

Lineage progression to establish skeletal muscle from a founder mesodermal cell in the embryo is common to all skeletal muscles. An undifferentiated mesodermal cell (fate is not acquired) will switch to a muscle progenitor state (fate being acquired) to finally end up as a differentiated muscle cell (functional entity). Such switches from an undifferentiated state to a fully differentiated state are regulated by the activation of different groups of transcription factors (Figure 4). Head, trunk and limb muscle progenitors are specified by different genetic programs, but once specified, myogenic cells use a common differentiation program.

In the body, myogenic specification requires *Pax3* and *Pax7* genes, belonging to the paired-box *Pax* family. PAX3 controls the delamination of epaxial myogenic progenitor cells (reviewed in Tajbakhsh and Buckingham, 2000). Moreover, the central domain of the dermomyotome gives rise to a PAX3/PAX7 progenitor population forming subsequent axial muscles. In *Pax3/Pax7* double-mutant mice, somitic cells do not enter the myogenic program, resulting in defective skeletal muscles (Kassar-Duchossoy et al., 2005; Relaix et al., 2005). The acquisition of a myogenic fate depends on a second group of transcription factors, named the basic Helix-Loop-Helix (bHLH) Myogenic Regulatory Factors (MRFs). MRFs have the ability to trigger skeletal muscle differentiation in non-muscle cells *in vitro* (Weintraub et al., 1991) and *in vivo* (Delfini and Duprez, 2004). *Myod1 (MyoD), Myf5*, and *Myf6* (*Mrf4*) are considered as the muscle determination factors (Kassar-Duchossoy et al., 2004), while *MyoG* (Myogenin) is associated with muscle differentiation (Hasty et al., 1993). However, both *Myod1* and *Myf6 (Mrf4)* are also required for terminal muscle differentiation (reviewed in Buckingham, 2006). *Myf5* and *Myf6* (*Mrf4*) regulate the entry of progenitor cells into the myogenic program when they delaminate from the lips of the dermomyotome to form the myotome, but subsequent hypaxial activation of *Myf5* is *Pax3*-dependent (Bajard et al., 2006). Early expression of *Myod1* depends on *Myf5, Myf6*, and *Pax3* as in the *Myf5/Myf6/Pax3* triple mutants, *Myod1* expression is altered and skeletal muscles do not form in the trunk and limbs (Tajbakhsh et al., 1997).

In vertebrates, the myogenic program of the head differs from the body musculature. While the expression of the myogenic regulatory factors *Myf5, Myod1*, and *Myog* in the craniofacial muscles is similar to what is observed in trunk/limb muscles (Hacker and Guthrie, 1998), the genetic hierarchies operating upstream of the myogenic genes are different for head muscles (branchiomeric and extraocular muscles). *Pax3* is not expressed in head muscles, and *Pax7* does not appear critical as head muscles form in the *Pax7* mutant mice (Relaix et al., 2004). While *Myf6* is not necessary for cranial myogenesis, other transcription factors among which *Tcf21* (*Capsulin*), *MyoR, Tbx1*, and *Pitx2* regulate the myogenic factors to form the different craniofacial muscles (Tzahor, 2009). *Tbx1* and *Pitx2* have been shown to activate *Myod1* and *Myf5* in the head and inactivation of *Tbx1* and *Pitx2* in mice causes severe reduction of specific groups of head muscles (Kelly et al., 2004; Zacharias et al., 2011). In mutant mice for *Tcf21* and *MyoR*, myogenic genes are not activated in branchiomeric muscles, and cells undergo cell death (Lu et al., 2002).

Myogenic factors are crucial intrinsic actors for correct development of muscle, however numerous studies have shown that their initiation and regulation also depends on secreted factors coming from the adjacent tissues. The influence of neural tube, neural crest cells, notochord and ectoderm on the formation of muscles has been previously extensively studied and showed that Shh, BMP, Wnt, FGF, and Notch signaling pathways participate to both axial and limb myogenesis (reviewed in Deries and Thorsteinsdóttir, 2016).

### Connective tissue-mediated muscle morphogenesis

CTs and muscles are closely related during embryonic development and adult stages, suggesting that interactions between these tissues might be essential for their development. Classical experiments in avian embryos have demonstrated that signals involved in muscle differentiation and patterning partly derived from surrounding tissues (Lance-Jones, 1988; Ordahl and Le Douarin, 1992; Kardon, 1998). Over the last years, thanks to the identification of specific molecular markers for the different types of CT, progress has been made in the dissection of mechanisms underlying the interactions between CT and muscle development. These data have shown that depending on their embryological origin and their position throughout the body, mechanisms and signaling pathways coming from the diverse types of CT influence spatially and temporally muscle morphogenesis.

### Specialized connective tissue-mediated myogenesis (bone and cartilage)

**Limb muscles** and specialized CTs (bone and cartilage) do not exhibit direct physical interactions, as they are linked together via tendons. During limb development, processes regulating skeleton and muscle formation can be dissociated (Hasson et al., 2010; Li et al., 2010). Indeed, disruption of skeletogenesis, through the mutation in the LIM-homeodomain transcription factor *Lmx1b* in skeletal progenitors using the *Sox9-Cre*, has no effect on muscle development (Li et al., 2010). Similarly, inactivation of the BMP antagonist, *Noggin*, which is expressed in condensing cartilage and immature chondrocytes, leads to profound skeletal defects without affecting the early stages of myogenic differentiation (Tylzanowski et al., 2006). However, despite the fact that skeleton and muscle formation can be dissociated, it has been evidenced that skeleton-derived signals are required for proper myogenesis. Indeed, although no defect at the onset of myogenesis is observed in *Noggin* null-mutant mice, terminal muscle differentiation is impaired (Tylzanowski et al., 2006; Costamagna et al., 2016). The Indian hedgehog (Ihh) secreted factor which belongs to the Hedgehog family is secreted by developing chondrocytes (Vortkamp et al., 1996). In the absence of Ihh, muscles are affected (Bren-Mattison et al., 2011). As for Noggin null-mutant, muscle impairment is restricted to secondary myogenesis, resulting in a decrease in the muscle masses. Finally, *in vitro* experiments show that C2C12 myoblasts can be converted toward osteogenic lineage when exposed to BMPs (Lee et al., 2000).

**Axial muscles** develop from the myotomal compartment of the somite, which is formed by the delamination of cells deriving first from the dorsomedial lip of the dermomyotome and then from its caudal and rostral lips. This process is partly controlled by another somatic compartment, the sclerotome. During chick embryonic development, pioneer myoblasts, constituting the medial part of epithelial somites, express the receptor ROBO2, while its ligand SLIT1 is expressed in the caudal domain of the nascent sclerotome (Halperin-Barlev and Kalcheim, 2011). Loss-of-function assays targeting either ROBO2 or SLIT1 lead to similar results: disruption of the caudo/rostral migration of pioneer myoblasts and of myofibre formation, demonstrating that skeletal precursor-derived signals (sclerotome) regulate the myotome morphogenesis (Halperin-Barlev and Kalcheim, 2011). However, since the sclerotome give rise to both skeleton and tendon progenitors (syndetome), these experiments cannot discriminate between the effects of bone or tendon progenitors on muscle morphogenesis.

Skeletal elements in the **head** derive from the cranial neural crest cells (Couly et al., 1992). Using a *HoxA1/HoxB1* double-knockout mouse, it was shown that cranial neural crest cells fail to form and migrate into the second branchial arch. Despite the absence of neural crest cells (at the origin of skeletal progenitors), cranial myogenesis was initiated (Rinon et al., 2007). However, muscle patterning defects were observed, as evidenced by the expansion in *Tcf21* (Capsulin) and *Tbx1* expression (Rinon et al., 2007). Similarly, ablation of cranial neural crest cells in the chick embryo shows that early steps of head myogenesis are not impaired by the removal of skeletal progenitors but that expression of myogenic genes is expanded to fill the entire arch mesenchyme, suggesting that the nature of the interactions between cranial skeleton and muscles are conserved in chick and mouse embryos (Tzahor et al., 2003; Rinon et al., 2007). Analysis of the molecular mechanisms demonstrate that BMP and Wnt signaling are important actors involved in these interactions (Tzahor et al., 2003; Rinon et al., 2007). However, cranial neural crest cells give rise to skeleton, tendon and CT progenitors. It is then difficult to determine in these experiments whether cranial myogenesis is controlled by interactions coming from prospective bone, tendon or muscle CT.

### Dense regular connective tissue (tendon) as an important source of signals during muscle development

Muscle and dense regular CT (tendon) displays interactions during their development. It is well established that tendon requires muscle to fully develop in chick, mouse and zebrafish embryos (reviewed in Gaut and Duprez, 2016). However, the influence of tendon on muscle development is less clear in vertebrates. During limb muscle development, muscle masses differentiate between tendon primordia. In experimentally tendon-depleted region in chick embryo, ectopic muscles form at the place where tendons normally develop, indicating the role of tendon in delimitating regions of muscle growth and differentiation (Kardon, 1998). The role of tendon cells on muscle development has been studied more in Drosophila. Drosophila tendon precursor cells are defined as a group of ectodermal cells, named the apodeme and characterized by the expression of the Early growth response (EGR)-like transcription factor *Stripe* (Frommer et al., 1996). Altering apodeme formation during the early steps of leg development affects the localization of myoblasts (Soler et al., 2016). Establishment of the myotendinous junctions also requires correct migration of myogenic cells toward tendon cells. This migration step is mediated through guidance cues delivered by tendon cells. In tendons, *Stripe* positively regulates the expression of the *Slit* gene (Volohonsky et al., 2007), coding for a secreted protein implicated in guidance cues during axonal migration (Wong et al., 2002). *Slit* is expressed by tendon cells, while its receptor *Robo* (Roundabout) is expressed in muscle (Kramer et al., 2001). Interestingly, *Slit* mutants present defects in muscle patterning (Ordan et al., 2015), revealing tendon-signaling requirement for proper muscle development. Tendon and muscle interactions via *Slit/Robo* is necessary for the migration arrest of muscle progenitors in Drosophila (Wayburn and Volk, 2009). The formation of the myotendinous junction in Drosophila also requires the transmembrane protein KON-TIKI, enriched at the tips of myotubes, and necessary to direct their migration and the subsequent recognition between muscle and tendon cells (Schnorrer et al., 2007). These data indicate that tendon cells are required for muscle morphogenesis through specific signals emanating from tendon cells and acting on myogenic cells. However, these signals remain to be elucidated during development of the vertebrate musculoskeletal system. In zebrafish, *Tsp4b* (thrombospondin-4) appears critical to orchestrate tendon extracellular matrix assembly necessary for muscle attachment at the myotendinous junction (Subramanian and Schilling, 2014). Although it has been shown that the vertebrate orthologs of *Stripe, Egr1/2* are involved in vertebrate tendon differentiation (Lejard et al., 2011; Guerquin et al., 2013), there is no obvious defect in muscle formation in the absence of *Egr1*. Inactivation of *Tsp4* in mice shows that thrombospondin-4 controls the deposition of extracellular matrix in both tendon and muscle and is necessary for the correct organization of collagen fibrils in tendon (Frolova et al., 2014). However, the absence of *Tps4* also directly affects skeletal muscle structure, by controlling the expression of heparan-sulfate proteoglycans in muscle (Frolova et al., 2014). Finally, tendons have been shown to be required in late events of vertebrate muscle morphogenesis. Indeed, the translocation of myofibers to form the final position of the flexor digitorum superficialis muscle in the mouse forelimb is largely impaired in *Scx* mutant, showing that tendon is implicated in the final patterning and position of muscles (Huang et al., 2013).

### Dense irregular connective tissue establish a pre-pattern for muscle differentiation

Most of our knowledge concerning myogenesis regulation by signals produced by the irregular CT has been established in the limb. Each step of limb muscle development is tightly regulated by signals among which some are derived from the irregular CT. The different steps are the following. Somitic-PAX3-positive cells migrate toward the limb bud, invading the limb mesenchyme. Once they reached their target sites, PAX-3 positive cells proliferate and organize into dorsal and ventral muscle masses. Muscle differentiation is then initiated, followed by muscle mass growth and splitting (reviewed in Duprez, 2002; Deries and Thorsteinsdóttir, 2016).

#### Delamination and migration of muscle progenitors

Delamination and migration of muscle progenitor cells from the ventrolateral lip of the dermomyotome are mediated via the tyrosine kinase receptor c-Met and its ligand, the Scatter Factor/Hepatocyte Growth Factor (SF/HGF), (Brand-Saberi et al., 1996; Heymann et al., 1996; Dietrich et al., 1999). Cells from the ventrolateral lip of the somite express *c-Met*, while SF/HGF is released by irregular CT progenitors in the limb mesenchyme. In *Hgf* or *c-Met* mutant mice, limb muscles are missing (Bladt et al., 1995). Dermomyotome development proceeds normally and migratory somatic precursors are correctly specified but they remain aggregated and fail to migrate toward limb buds (Dietrich et al., 1999). SF/HGF also regulates the migration of myogenic progenitors from occipital and cervical somites, giving rise to the tongue, diaphragm and shoulder muscles (Dietrich et al., 1999). These studies highlight the link between irregular CT and hypaxial muscle progenitors during the migration step of muscle morphogenesis. Other signaling pathways expressed in irregular CT are involved in the guidance of muscle progenitors to reach their target sites into the limb bud. The CXCL12 chemokine is expressed in restricted regions of limb bud irregular CT and has been shown to attract muscle progenitors, which expressed the chemokine receptor CXCR4 (Vasyutina et al., 2005). Ectopic expression of CXCL12 in limb mesenchyme of chick embryos, or inactivation of *Cxcr4* in mouse embryos both give rise to aberrant localization of muscle progenitors in the limb (Vasyutina et al., 2005), demonstrating a chemoattractive role of CXCL12 positive-CT cells for *Cxcr4* expressing muscle precursors. During their migration toward the limb, muscle progenitors also express the receptor *EPHA4*, while its ligand *EPHRINA5* is expressed in specific areas of limb irregular CT (Swartz et al., 2001). Conversely to the chemoattractive role of CXCL12/CXCR4 signaling, EPHRINA5 acts as a repulsive signal for muscle cells expressing EPHA4 (Swartz et al., 2001), demonstrating that both chemoattractive and repulsive signals from irregular CT act simultaneously on muscle progenitors to restrict and define their pathway of migration. Finally, it is important for muscle progenitor cells to stay in an undifferentiated state during migration. It is likely that this step is regulated through secreted signals produced by the limb mesenchyme, however it is not clear yet which signaling exactly is involved in this process. Previous studies suggest that BMPs and FGFs secreted by limb irregular CT might be important to prevent differentiation in migrating cells by respectively inhibiting and promoting the expression of SF/HGF (Heymann et al., 1996; Pourquié et al., 1996; Scaal et al., 1999). In the chick embryo, FGF18 and retinoic acid, secreted by limb mesenchyme, control the timing of *Myod1* and *Myf5* expression in myogenic cells (Mok et al., 2014).

#### Muscle differentiation and patterning

During the whole processes of limb muscle morphogenesis, irregular CT and muscles (progenitors or differentiated cells) are in close association. Kardon et al. (2003) identified TCF4 as a putative actor in the process of irregular CT-mediated muscle morphogenesis. TCF4 is expressed in the lateral plate-derived mesoderm in close association with limb muscles during their differentiation and patterning. In the absence of limb muscles, TCF4 expression pattern is unchanged, suggesting that TCF4 expression may serve as a pre-pattern for limb musculature. To verify this hypothesis, *TCF4* gain- and loss-of-functions were performed in lateral plate-derived limb mesodermal cells. In all cases, muscle mispatterning was observed, demonstrating that TCF4 in irregular CT is important to establish the correct location of limb muscles (Kardon et al., 2003). *Tcf4* deletion in mice also lead to aponeurosis defects (Mathew et al., 2011). However, TCF4 is also expressed at low level in myogenic cells and is involved in the intrinsic regulation of muscle fiber type differentiation in mice (Mathew et al., 2011).

Recently, the role of irregular CT has also been involved in the context of a common and often lethal muscle diaphragm defect, called congenital diaphragmatic hernia (CDH). Merrell et al. (2015) have shown that the pleuroperitoneal folds, which are transient embryonic structures, give rise to the diaphragm irregular CT. Muscle progenitor cells arising from the ventrolateral dermomyotome of the cervical somites migrate into the *Tcf4*-positive pleuroperitoneal cells which guide muscle cells to organize the diaphragm morphogenesis. *Tcf4*-positive CT cells also express *Gata4*, known to be mutated in CDH, and Gata4 inactivation in diaphragm CT leads to hernias similar to those observed in CDH, demonstrating that this congenital muscular disease is related to a defect in muscle irregular CT (Merrell et al., 2015).

As previously mentioned, the human Holt-Oram syndrome is characterized by limb and heart musculoskeletal defects and irregular CT disorganization. This syndrome is due to a mutation in the *TBX5* gene, which is expressed in irregular CT during limb development (Hasson et al., 2010). *Tbx5* deletion leads to a defect in irregular CT organization during embryonic development (Hasson et al., 2010). In these conditions, while the early steps of limb myogenesis are not affected, ectopic splitting of nascent muscle bundles is observed. *Tbx5* inactivation leads to a disruption of muscle irregular CT, to an alteration of *Tcf4* expression, but also a marked decrease of ß-catenin and N-cadherin at the membranes of muscular irregular CT cells (Hasson et al., 2010). In addition, deletion of ß-catenin in the limb mesenchyme leads to ectopic muscle splitting consistent with a model in which the N-cadherin/ß-catenin complex in the muscle CT is critical for muscle patterning (Hasson et al., 2010). Finally, *Tbx5* deletion also alters the expression of mesenchymal secreted factors important in limb myogenesis, as CXCL12 and SF/HGF (Hasson et al., 2010). It is noteworthy that in synovial fibroblasts, *Cxcl12* is a target of *Tbx5* in human synovial fibroblasts (Karouzakis et al., 2014). Recently, it has been shown that the conditional deletion of another T-box gene, *Tbx3*, in the lateral plate mesoderm (using a Prx1-Cre transgene) leads to defects in myofiber formation in a subset of limb muscles in mice (Colasanto et al., 2016). These localized muscle defects are correlated with *Tbx3* expression in a subset of limb bones, tendons and muscle CT. Similar muscle defects are observed in patients with *TBX3* mutations that are responsible of the Ulnar-mammary syndrome (Colasanto et al., 2016). In addition to being expressed in limb skeletal elements, *Hoxa11* gene is also expressed in mouse muscle CT and *Hoxa11* inactivation disrupts limb muscle and tendon patterning in addition to the already known skeleton defect (Swinehart et al., 2013). Tendon and muscle phenotypes in heterozygous *Hoxa11* mutants are independent of skeletal patterning as abnormal tendon and muscle patterning are observed in *Hoxa11* mutants with normal skeleton (Swinehart et al., 2013). However, it cannot be excluded that, in this case, muscle mispatterning could be related to tendon abnormalities rather than to the muscle CT defect. Recently, Gu et al. (2016) have shown that in neonatal muscles, muscle interstitial cells activate NF-kB, which regulates EPHRINA5 to stimulate myoblast migration toward the end of growing fibers, where they subsequently fuse to contribute to muscle growth. These data show that muscle CT also contributes to the process of muscle maturation during neonatal development. However, these interstitial cells are characterized by the expression of NG2, a neural/glial antigen 2 expressed in pericytes and it cannot be excluded that these cells are of vascular origin (Gu et al., 2016).

Finally, differentiated muscle fibers also act on muscle CT formation. In mice deleted for *Lox* (Lysyl-oxidase), an enzyme regulating collagen organization and secreted from the myofibers, TGFβ signaling is decreased and promotes muscle CT differentiation at the expense of muscle tissue (Kutchuk et al., 2015).

### Connective tissue cell involvement in adult muscle homeostasis

In adult, skeletal muscle loss is observed in neuromuscular diseases, but also during aging, inactivity and chronic systemic disorders (i.e., diabetes, cancer, rheumatoid arthritis). The regenerative potential of skeletal muscle provides a compensatory response against such pathological muscle loss. The regenerative capacity of skeletal muscle relies on muscle stem cells (named satellite cells), which proliferate in response to exercise to facilitate muscle growth and remodeling, or following myotrauma to repair the injured muscle. Satellite cells are PAX3/7-positive progenitor cells located under the basal lamina that forms around muscle fibers of postnatal skeletal muscle. Satellite cells remain quiescent until the muscle is injured, when the lamina breaks down and activated satellite cells begin to proliferate before forming new muscle fibers (Relaix et al., 2005). *Myf5* is detected in the majority of quiescent satellite cells (Cornelison and Wold, 1997; Beauchamp et al., 2000) and activation of satellite cells is accompanied by expression of *Myod1* as well as higher levels of *Myf5*, leading to the downregulation of *Pax7*, activation of Myogenin, and new muscle fiber formation (Relaix et al., 2006, reviewed in Motohashi and Asakura, 2014). In the absence of *Pax7*-positive cells, the process of muscle regeneration failed and instead, fibrotic and fatty infiltration are observed, demonstrating the major contribution of muscle satellite cells in the formation of new muscle fibers (von Maltzahn et al., 2013). However, in response to muscle damage, non-myogenic cells can also participate to skeletal muscle regeneration, either by giving rise to myogenic stem cells or by stimulating the activation of resident muscle satellite cells.

A non-satellite cell population with myogenic capacity was first identified when it has been shown that bone-marrow-derived cells can participate directly to muscle regeneration (Ferrari et al., 1998). These cells, which normally reconstitute the hematopoietic lineage, can give rise to new satellite cells and myofibers during the muscle regeneration process (Asakura, 2012) and their transplantation into mdx mice (a model for Duchenne muscular dystrophy) improves muscle function (Sampaolesi et al., 2006). Similarly, a vascular progenitor population, which can be isolated from postnatal muscle, participate in muscle repair following arterial delivery in mice (Sampaolesi et al., 2003). Interestingly, pre-treatment of both mesenchymal bone-marrow stromal cells (Galvez et al., 2006) or vascular progenitors (Brzoska et al., 2012) with the CXCL12 chemokine improved the regeneration of injured muscle. CXCL12 is expressed in the adult muscle by the endomysium, i.e., the CT surrounding each muscle fiber (Hunger et al., 2012). Following muscle injury, CXCL12 secreted by muscle CT rapidly increases (Griffin et al., 2010) and chemoattracts both satellite cells and bone-marrow-derived cells to actively participate to the regeneration process (Ratajczak et al., 2003). In this context, CXCL12 would not only chemoattract stem cells toward the injury site, but would also increase their fusion with native muscle fibers (Griffin et al., 2010). These results demonstrate that signals provided by muscle irregular CT are not only crucial for muscle morphogenesis during development but also mediate the processes of muscle regeneration in the adult.

More recently, a population of interstitial muscle cells with myogenic potential has been identified (Mitchell et al., 2010). These cells, characterized by the expression of the PW1/*Peg3* gene and named PICs (PW1-positive interstitial cells) contribute to the satellite cell pool during muscle regeneration (Mitchell et al., 2010), although they do not express Pax3 or Pax7 (Pannérec et al., 2013). PICs can be subdivided into two distinct populations: PW1+ PDGFrα− and PW1+ PDGFrα+ cells. It has been establish that only PW1+ PDGFrα− PICs are associated with a myogenic potential while PW1+ PDGFrα+ cells give rise to adipocytes (Pannérec et al., 2013). Interestingly, PW1+ PDGFrα+ PICs express the pericyte marker NG2, indicating a possible overlap between these cells, and pericytes (Pannérec et al., 2013). Pericytes represent perivascular cells that are present in the muscle interstitium and associated with capillaries. They can be separated into two different populations: type-1 pericytes (NG2+ NESTIN− PDGFrα−) and type-2 pericytes (NG2+ NESTIN+ PDGFrα+), (Birbrair et al., 2013). Similarly to what has been described for PICs, the two different populations of pericytes have distinct cell fate potential: type-1 contribute to adipose tissue and type-2 to myogenesis (reviewed in Birbrair et al., 2014). Type-2 pericytes do not express Pax7, Myf5 and Myod1, but upregulate these markers before forming myotubes in regenerative conditions (Cappellari and Cossu, 2013).

Different studies also reported the participation of mesenchymal progenitors without myogenic capacity during muscle regeneration. These progenitors all arise from resident cells in the adult muscle interstitium (Joe et al., 2010; Uezumi et al., 2010). Based on the expression of PDGFRα, a cell population resident in the muscle interstitium has been isolated, which, under specific culture conditions, differentiate into fibroblasts, adipocytes or osteoblasts, but never give rise to muscle cells and has been named mesenchymal progenitors (Uezumi et al., 2010). Simultaneously, Rossi's group also identified a cell population with fibroblastic and adipogenic potential, but no myogenic potential (Joe et al., 2010). These progenitors were isolated on the basis of SCA1 and CD34 expression, and termed Fibro/Adipogenic progenitors (FAPs), (Joe et al., 2010). Interestingly, mesenchymal PDGFRα+ progenitors express SCA1 (Joe et al., 2010, Uezumi et al., 2010) and FAPs express PDGFRα, highlighting the possibility that mesenchymal progenitors and FAPs actually represent a unique progenitor population. FAPs/mesenchymal progenitors are activated upon muscle injury and promote myoblast differentiation in co-cultures (Joe et al., 2010), but also exhibit a strong adipogenic and fibrogenic potential *in vitro*, indicating a potential contribution of FAPs to fibrotic and adipose accumulation in diseased muscles (Uezumi et al., 2010). It is then proposed that a balance between satellite cell-dependent myogenesis and FAPs-dependent adipogenesis/fibrogenesis regulates muscle homeostasis and regeneration. After muscle injury, FAPs/mesenchymal progenitors start to proliferate before satellite cells and invade the space between regenerating muscle fibers, where they generate factors promoting myogenesis. When regeneration proceeds efficiently, FAPs/mesenchymal progenitors are discarded from the tissue through apoptotic signals emanating from satellite cells. If regeneration fails, FAPs/mesenchymal progenitors persist and differentiate into adipocytes and fibroblasts, leading to fatty and fibrotic degeneration (reviewed in Natarajan et al., 2010; Judson et al., 2013). Depending on the surrounding environment, FAPs/mesenchymal progenitors will preferentially give rise to fibroblasts or adipocytes. Addition of TGFß to FAPs/mesenchymal progenitors *in vitro* induces the expression of fibrosis markers leading to fibroblastic differentiation at the expense of adipocyte differentiation (Uezumi et al., 2011). Interestingly, PDGFRα+ expressing FAPs/mesenchymal progenitors accumulate preferentially into fibrotic regions, suggesting a specific role for PDGFRα in muscle fibrosis (Uezumi et al., 2014). This hypothesis is supported by the observation that, in adult and embryonic mouse, an elevated level of PDGFRα leads to an abnormal increase in CT differentiation (Olson and Soriano, 2009).

The participation of irregular CT to muscle regeneration has been also highlighted by a recent set of experiments. CT fibroblasts identified by *Tcf4* expression have been shown to proliferate close to muscle satellite cells following injury and conditional ablation of *Tcf4*-positive cells prior to muscle lesion leads to premature satellite cell differentiation, depletion of the early pool of satellite cells, and small regenerated fibers, indicating that *Tcf4*-positive fibroblasts participate in muscle regeneration (Murphy et al., 2011). It remains unclear whether a direct relationship exists between FAPs/mesenchymal progenitors and TCF4-positive cells. However, *Tcf4*-positive cells express PDGFRα (Murphy et al., 2011) and accumulating evidence suggests that FAPs/mesenchymal and irregular CT progenitors share common features (Sudo et al., 2007; Haniffa et al., 2009). Extracellular matrix components also contribute directly to the regenerative potential of muscle. Indeed, it has been shown that a fibronectin-rich fibrosis is essential during the initial step of regeneration to activate the proliferation of muscle satellite cells (Bentzinger et al., 2013). Irregular CT progenitors, FAPs and PICs could be potential sources of fibronectin and might contribute to the transient fibronectin-rich promyogenic fibrosis during muscle regeneration. However, activated satellite myogenic cells themselves release fibronectin into their microenvironment and inactivation of fibronectin using a *Myf5*-Cre reporter impairs the regenerative potential of muscle, suggesting that this effect could be also related to a cell-autonomous role of satellite cell derived-fibronectin (Bentzinger et al., 2013).

The importance of muscle CT has been also evidenced in muscle disorders. Indeed, mutations in *COL6A1, COL6A2*, and *COL6A3* genes, which give rise to the main collagens expressed in muscle CT, have been observed in congenital Ullrich muscular dystrophy and in Behlem myopathy. Mutant mice for *Col6a1* display alterations of muscle sarcoplasmic reticulum and mitochondria (Pan et al., 2014) and *Col6a3* mutant mice display myopathic and connective tissue phenotypes similar to the *Col6a1* null mice (Pan et al., 2013), demonstrating that collagen VI mutations result in disorders with combined muscle and connective tissue involvement. In addition, *Col6a1* mutant mice showed delayed muscle regeneration and reduced satellite cell self-renewal. Transplantation of wild-type fibroblasts in muscles of *Col6a1* mutant mice rescues muscle satellite cells, indicating that COL6A1 in the muscle environment can modulate satellite cell behavior (Urciuolo et al., 2013).

Finally, during muscle hypertrophic activity, satellite cells can regulate fibrogenic cell collagen expression via exosome secretion, showing that muscle cell progenitors can also act with their surrounding environment to facilitate tissue plasticity (Fry et al., 2016). Similarly, Abou-Khalil et al. (2015) have shown that *Pax7*-positive muscle satellite cells are involved in bone repair, providing a direct evidence of a muscle contribution to specialized CT (bone and cartilage) formation.

Taken together, these data evidence interactions between different cell populations promoting muscle progenitor activation during regeneration, with a central role of muscle irregular CT in this process. Changes in CT local environment may contribute to muscle pathologies and age-related loss of muscle stem cell competence by implicating pivotal signaling pathways and genes similar to those described to mediate the CT-dependent muscle morphogenesis during development.

## Conclusions

The development of skeletal muscle has been extensively studied for decades and most of the studies have first concentrated on the elucidation of the intrinsic mechanisms underlying the conversion of muscle progenitors toward a functional skeletal muscle organ. The identification of specific myogenic transcription factors has allowed us to decipher the importance of these intrinsic gene networks in the specification and differentiation of muscles during embryonic development. In parallel, the role of neighboring tissues on muscle morphogenesis has been investigated and highlighted the influence of the neural tube, notochord and ectoderm on the early steps of axial muscle morphogenesis, mostly via the effect of the secreted factors Wnts, BMPs, and SHH. More recently, the role of CTs in muscle morphogenesis has been investigated, thanks to the identification of transcription factors specifically expressed in the different types of CT surrounding (tendon) or composing (muscle CT) the developing muscle (previously reviewed in Hasson, 2011). These studies demonstrate that mesenchymal cells at the origin of the different CT types deliver information necessary for a correct muscle morphogenesis, from the early steps of myoblast migration and fusion to the late stages of muscle maturation. Secreted factors as BMPs, FGFs, and chemokines as CXCL12 are important in this dialogue between CTs and muscles, which also implicate a reverse interaction between both tissues, as muscle cells are necessary for the tendons to develop correctly and for the organization of irregular CT and bone in adult. A right balance between myogenic and CT cells is particular necessary during the muscle regeneration process. Indeed, impairment of the regenerative potential after injury or in neuromuscular diseases results in the progressive replacement of the muscle mass by fibrotic tissue (Farup et al., 2015). Thus, bi-directional communication between muscle and CT is critical for a correct assembly of the musculoskeletal system during development as well as to maintain its homeostasis in the adult.

## Author contributions

SN, DD, and CF have equally contributed to making the original plan and writing the manuscript.

## Funding

This work was supported by the Fondation pour la Recherche Médicale (FRM) DEQ20140329500, Agence Nationale de la Recherche (ANR) ANR-12-BSV1-0038, Association Française contre les Myopathies (AFM) N°16826. SN was part of the MyoGrad International Research Training Group for Myology and received financial support from the AFM (AFM 20150532272).

### Conflict of interest statement

The authors declare that the research was conducted in the absence of any commercial or financial relationships that could be construed as a potential conflict of interest.
